# NK cell subsets and dysfunction during viral infection: a new avenue for therapeutics?

**DOI:** 10.3389/fimmu.2023.1267774

**Published:** 2023-10-19

**Authors:** Jacob C. Bjorgen, Jenna K. Dick, Ross Cromarty, Geoffrey T. Hart, Joshua Rhein

**Affiliations:** ^1^ Division of Infectious Diseases and International Medicine, Department of Medicine, University of Minnesota, Minneapolis, MN, United States; ^2^ Masonic Cancer Center, University of Minnesota, Minneapolis, MN, United States; ^3^ Center for Immunology, University of Minnesota, Minneapolis, MN, United States

**Keywords:** NK cells, NK cell dysfunction, HIV, hepatitis C, CMV, influenza, SARS-CoV-2, immunotherapy

## Abstract

In the setting of viral challenge, natural killer (NK) cells play an important role as an early immune responder against infection. During this response, significant changes in the NK cell population occur, particularly in terms of their frequency, location, and subtype prevalence. In this review, changes in the NK cell repertoire associated with several pathogenic viral infections are summarized, with a particular focus placed on changes that contribute to NK cell dysregulation in these settings. This dysregulation, in turn, can contribute to host pathology either by causing NK cells to be hyperresponsive or hyporesponsive. Hyperresponsive NK cells mediate significant host cell death and contribute to generating a hyperinflammatory environment. Hyporesponsive NK cell populations shift toward exhaustion and often fail to limit viral pathogenesis, possibly enabling viral persistence. Several emerging therapeutic approaches aimed at addressing NK cell dysregulation have arisen in the last three decades in the setting of cancer and may prove to hold promise in treating viral diseases. However, the application of such therapeutics to treat viral infections remains critically underexplored. This review briefly explores several therapeutic approaches, including the administration of TGF-β inhibitors, immune checkpoint inhibitors, adoptive NK cell therapies, CAR NK cells, and NK cell engagers among other therapeutics.

## 1 Introduction

Natural killer (NK) cells are a type of lymphocyte notable for their cytotoxic activity and role as an innate immune cell that acts as an early responder to physiologically stressed cancer and virally infected cells (1). NK cells are particularly important in viral infections, given their ability to respond to them before the development of adaptive immune responses (2). Evidence for a role of NK cells in the protection against certain viral infections is appreciated in patients with selective NK cells deficiencies; the hallmark clinical feature in these patients are severe and recurrent viral infections (3, 4). For example, of a group of 19 NK cell deficient patients, 42% died prematurely while 53% had experienced severe herpesvirus infections including cytomegalovirus, Epstein-Barr virus, Varicella Zoster virus, and herpes simplex virus (5). The dramatically increased incidence rate of severe complications related to these infections in patients with NK cell deficiency is strongly illustrative of their important role in combating and containing viral infections.

### 1.1 NK cell subsets

Like other immune cells, NK cells originate from CD34^+^ hematopoietic stem cells, and most of their development occurs in discrete stages. Commitment to the NK cell lineage relies on loss of CD3 and gain of the natural cytotoxicity receptors NKp44 and NKp46 (6). Roughly 5-15% of all circulating lymphocytes is made up of NK cells, and NK cells are known to also reside in secondary lymphoid tissue, bone marrow, lungs, liver, spleen, and uterine tissues.

NK cells have been divided into different populations based on the relative expression of surface markers CD56 and CD16 (FcgrIIIa), with the two largest subsets being CD56^bright^ and CD56^dim^ (7). CD56^bright^ NK cells constitute roughly 2-10% of all NK cells in the blood and are found mainly in the secondary lymphoid organs where they constitute 50% of the NK cells, whereas CD56^dim^ NK cells comprise 90% of NK cells in the blood (8). CD56^bright^ NK cells differentiate into CD56^dim^ NK cells and then express CD16 (9, 10). Functionally, CD56^bright^ NK cells are considered to be far less cytotoxic than their CD56^dim^ counterparts, and play a role in the production of antiviral proinflammatory cytokines like IFN-γ and TNF-α (7, 8). In contrast, CD56^dim^ NK cells are far more cytotoxic since they contain 10-fold more perforin and Granzyme B and due to their expression of CD16, which enables them to engage in antibody-dependent cellular cytotoxicity (ADCC) (11). CD16 is the only receptor known to activate NK cells on its own without activation through other receptors (12). Of note, CD56^dim^ NK cells are also capable of producing proinflammatory cytokines like IFN-γ, typically producing them two to four hours after stimulation, whereas CD56^bright^ NK cells predominantly produce cytokines 16 hours or more after stimulation (13). There is another subset of NK cells, termed adaptive (or memory-like) NK cells, that possess qualities of the adaptive immune system such as antigen-driven clonal expansion and long lived memory that have been recognized and will be examined in more detail later (14). Finally, there are also different subsets of NK cell populations that reside in peripheral tissues that carry distinct phenotypic markers (15).

### 1.2 NK cell receptors and signaling

**Figure 1:**
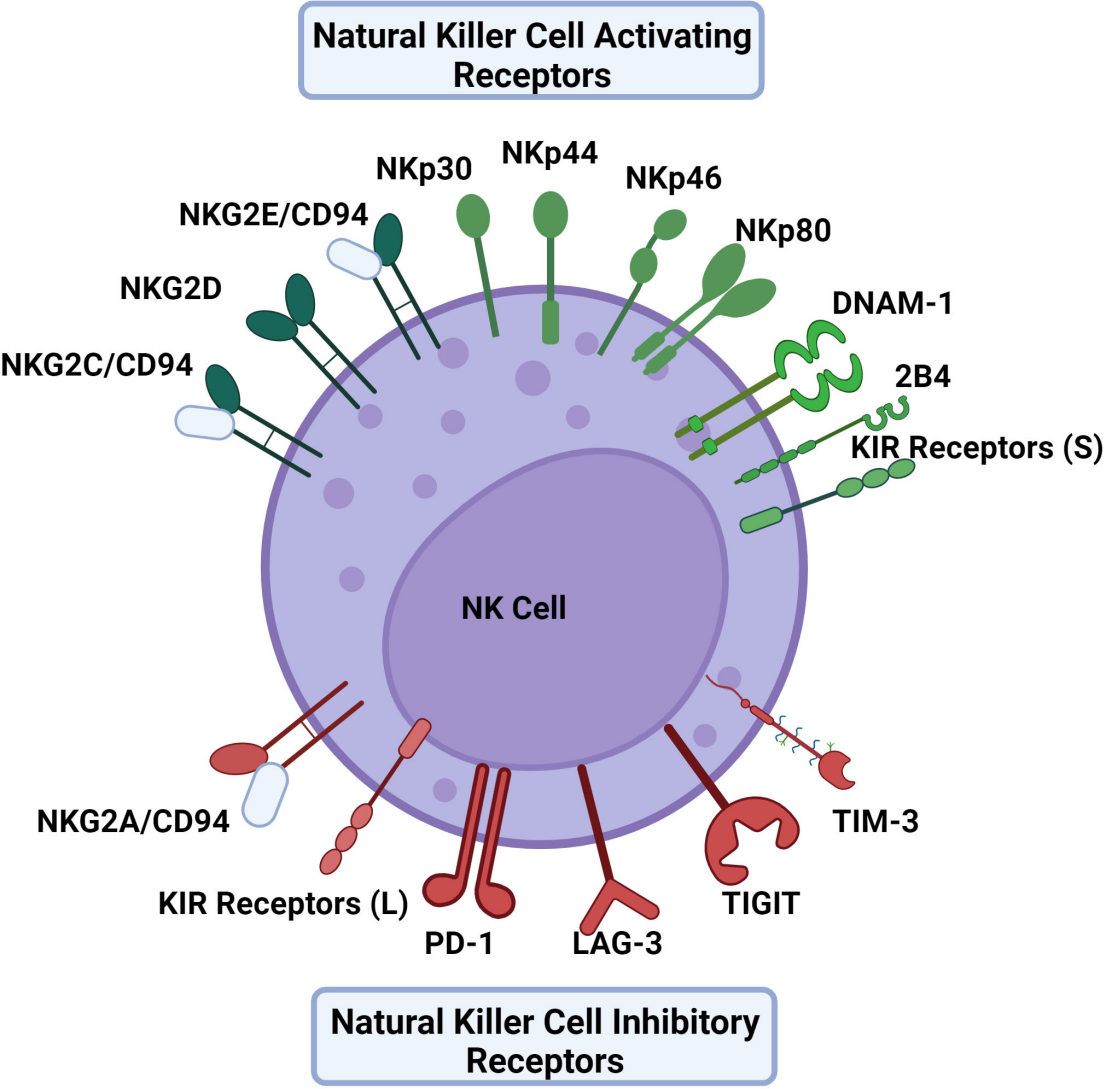
The activating and inhibitory receptors of NK cells. NK cell activation depends on a balance of activating and inhibitory signals using receptors that primarily bind HLA to distinguish between virally infected cells and uninfected cells in a non-antigen specific mechanism. Activating receptors include NKG2C, NKG2D, NKG2E, NKp30, NKp44, NKp46, NKp80, DNAM-1, and KIR receptors with short cytoplasmic tails. Inhibitory receptors include NKG2A, PD-1, LAG-3, TIGIT, TIM-3 and KIR receptors with long cytoplasmic tails excluding KIR2DL4 which is activating. The KIR receptor family contains both activating and inhibitory receptors, as well as receptors that can act as either. Similarly, 2B4 can also provide either an activating or inhibitory signal.

NK cells are unique in their mechanism of recognition of dysregulated or infected cells. Instead of solely recognizing viral peptides presented by major histocompatibility (MHC) molecules like cytotoxic T lymphocytes, NK cell activation is dependent on the balance between different activating and inhibitory receptors to distinguish between virally infected and uninfected cells in a non-antigen-specific manner. Many NK cell receptors sense surface expression of MHC class I molecules as well as MHC class I chain related (MIC) molecules among others (16). NK cell activation will only occur if the activating signal overrides the inhibitory signal (**Figure 1**) (17). Importantly, NK cells can recognize virally infected cells or tumor targets without prior sensitization to antigens. Some viruses will down-regulate MHC-I to interfere with the presentation of viral antigens, thereby escaping cytotoxic T lymphocytes. This downregulation of MHC-I promotes the recognition and killing of virally infected cells by NK cells. This concept of recognizing cells that lack MHC-I is known as the “missing-self” hypothesis (18).

Activating receptors include natural cytotoxicity receptors (NCRs) such as NKp30, NKp44, NKp46, and NKp80, in addition to some receptors of the NKG2 family, including NKG2C, NKG2D, and NKG2E (7, 19). Resting NK cells can express NKp30, NKp46, and NKp80 whereas NKp44 is expressed by activated NK cells (20). The acquisition of natural cytotoxicity receptors during NK cell maturation is correlated with the development of cytotoxic activity against tumor cells depicting the receptor family’s importance in NK cell activation (21). NKG2C and NKG2E form heterodimers with CD94 which bind to HLA-E, an interaction that supports NK cell expansion in antiviral responses (17). Another activating receptor is DNAM-1 which binds nectin and nectin-like molecules (22).

Inhibitory receptors include the members of the killer cell immunoglobulin-like (KIR) receptor family that have long cytoplasmic tails (23). Of note, while many KIR receptors are inhibitory, a portion are activating and have short tails, and some can be both activating and inhibitory (24). Similarly 2B4 is another NK cell receptor that has been observed to be both activating and inhibitory depending on the presence of modulating co-receptors and signaling molecules (25). NKG2A/CD94 heterodimers also serve as another important inhibitory receptor that binds HLA-E as its ligand (26). In the blood, approximately 50% of NK cells express NKG2A/CD94 receptors (27). In terms of NK cell differentiation, it has been noted that the NK cell maturation process entails a stepwise decline in NKG2A with a simultaneous increase in killer immunoglobulin-like receptors (KIRs) (28). TIGIT, LAG-3, TIM-3, and PD-1 receptors are classically defined as inhibitory receptors on cytotoxic T lymphocytes, but can serve a role in modulating NK cell responses in an inhibitory or activating fashion depending on the context (29, 30).

### 1.3 Genetic variation in NK cell receptors and ligands

Adding to the complexity of NK cell signaling, extensive genetic diversity exists within many NK cell receptors and ligands, particularly within the KIR receptor family and HLA genes (31–33). To elaborate, the KIR receptor family has been found to vary widely in terms of the presence of different KIR genes, copy number of KIR genes, and there exsists substantial polymorphism within these genes resulting in markedly different KIR repertoires between individuals (34, 35). Two primary haplotypes have been identified and termed group A and group B (36). The group A haplotype is relatively consistent in its makeup, consisting of the activating KIR receptor KIR2DS4 and the inhibitory KIR receptors KIR2DL1, KIR2DL3, KIR3DL1, KIR3DL2, and KIR2DL4 (31). In comparison, the B haplotype is less fixed and contains one or more activating KIR receptor along with genes encoding the inhibitory KIR receptors KIR2DL5A, KIR2DL5B, and KIR2DL2. Within haplotypes, group A varies in terms of allelic polymorphisms in its constituent genes, while group B varies more in terms of gene content and copy number, although allelic variation is also a consideration too (36). The HLA gene family, whose constituents serve as ligands for many NK cell receptors encompasses more than 35,000 different MHC alleles, making it the most polymorphic region in the human genome (37). The implications of this diversity on NK cell signaling are made very apparent by the observation that various combinations of KIR receptors and HLA ligand have displayed differential functional activation (32, 38–40), and have additionally been associated with infection outcome in human immunodeficiency virus (HIV) (41, 42), hepatitis C virus (HCV) (43), SARS-CoV-2 (44), and ebola virus (45).

A similar effect can be observed from the effect of genetic variation within the NKG2D/NKG2DL signaling axis. While NKG2D is evolutionarily conserved relative to the KIR receptor family, distinct haplotypes exist and have been implicated in predisposing different cancer risk (46), as well as being a prognostic factor for treatment free remission in the setting of chronic myeloid leukemia (47). In humans, the ligands for NKG2D are MIC and ULBP which are upregulated by virally infected cells, and both of these ligands display extensive polymorphism (48). Certain alleles in these ligands have been associated with diseases like Dengue shock syndrome (49, 50), colorectal cancer (51), oral squamous cell carcinoma (52, 53), breast cancer (54), and cervical cancer (55). Taking the examples of the KIR receptor family and NKG2D/NKG2DL axis together, it is very evident that the genetic content of an individual's NK cell repertoire plays a significant role in the regulation of NK cell function in disease.

### 1.4 Additional NK cell markers

Other notable NK cell markers include CD57, CD69, and CD107a. CD57 expressing NK cells are connected with terminal differentiation, senescence, and high cytotoxic potential (56). CD69 is a marker expressed by NK cells during acute activation (57, 58). Additionally, CD69 has been shown as a marker of tissue resident NK cells (59). CD107a is expressed on the inner membrane of cytotoxic granules containing molecules like granzymes and perforin, which are released upon NK cell activation. When the granule is released, its membrane fuses with the outer cell membrane making cells that released granules positive for CD107a. Therefore, CD107a as a marker is indicative of NK cell granule release (degranulation) (60).

NK cells primarily kill infected cells through the release of granules containing granzyme-B and perforin, a serine protease that activates apoptosis and a protein that forms pores in the membrane of targeted cells, respectively (61, 62). In this process, perforin enables granzymes to infiltrate past the membrane of target cells, where they cleave internal components including procaspases that then initiate apoptosis (63). ADCC is another important mechanism through which NK cells kill target cells. In ADCC, the target cell is bound by antibodies and the Fc region of these antibodies in turn binds CD16, ultimately triggering the release of cytotoxic effector molecules that kill the target cell as described above (64).

During the NK cell response to viral infection, the repertoire of NK cell activating and inhibitory receptors is altered, often with an impact on the functionality of these cells. The aim of this review is to summarize these changes, particularly those related to NK cell dysregulation and exhaustion in the context of HIV, HCV, influenza A viruses (IVA), cytomegalovirus (CMV), and SARS-CoV-2. These viruses were chosen due to their clinical relevance and the existence of a plethora of literature noting alterations in NK cell phenotype and repertoire during their pathogenesis. Each of these viruses represent different viral families, and furthermore, the selection offers perspective into the similarities and differences between acute and chronic viral infections’ impact on the NK cell population. Further, potential therapeutics that could help maintain, or enhance an effective NK cell response will be explored.

## 2 NK cells during viral infection

### 2.1 Human Immunodeficiency Virus (HIV)

HIV belongs to the Retroviridae family and has a genome that consists of two single-stranded RNA molecules (65). HIV primarily targets CD4^+^ T cells and ultimately results in their depletion rendering those infected progressively immunocompromised and susceptible to opportunistic infections and rare forms of cancer (66). With contemporary antiretroviral therapy (ART), replication of the virus can be controlled to the extent that patients on ART are aviremic (66). However, ART is not curative due to the establishment of reservoirs of infected cells that persist in lymphoid tissue and are often latent (67). Significant attention has been placed on characterizing the role of NK cells in HIV infection within both the blood and lymphoid tissue given the nature of the infection.

**Figure 2:**
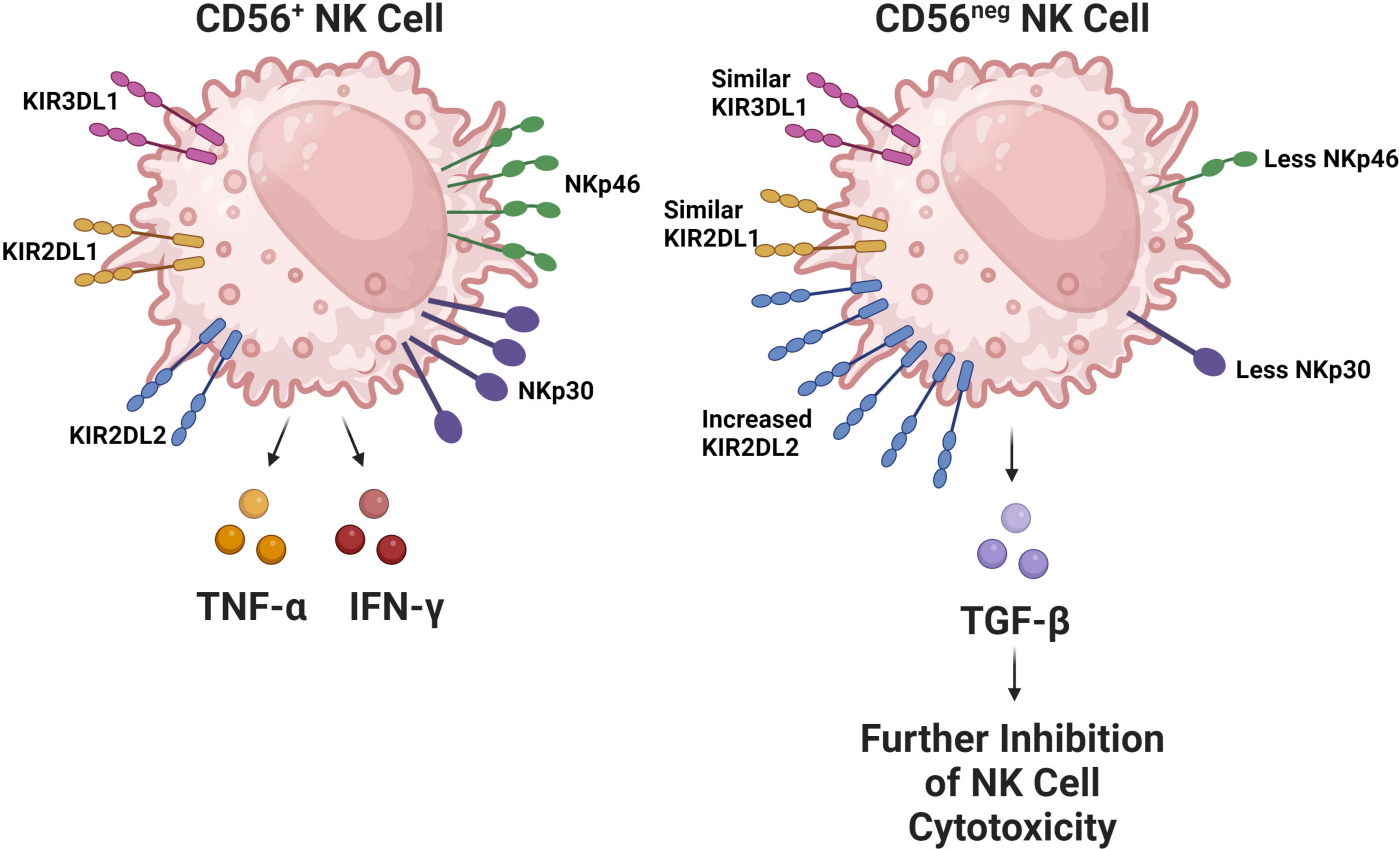
The CD56^neg^ NK cell subset increases during chronic viral infections. This NK cell subset has been observed to expand in the setting of HIV, chronic HCV, EBV, and chronic CMV. The subset has substantially impaired cytolytic capabilities due to alterations in its receptor repertoire, particularly due to decreased expression of activating receptors like NKp30 and NKp46 in addition to the increased expression of the inhibitory KIR receptor KIR2DL2 (52). Interestingly, the expression of inhibitory KIR receptors KIR2DL1 and KIR3DL1 remain comparable between CD56^+^ and CD56^neg^ NK cells. In HIV, this subset has been found to be more expressive of TGF-β, which contributes to the exhaustion of other NK cells.

In the context of HIV infection, and the general immune dysregulation incurred by depletion of CD4 T cells, NK cells undergo significant changes regarding their receptor expression and functional activity. HIV progression in terms of declining CD4 T cell counts is associated with a decline in the quantity of cytotoxic CD56^dim^ NK cells (68, 69). The remaining CD56^dim^ NK cell population has CD57 and KIR receptor expression characteristic of a more mature phenotype. However, these cells have decreased functional potential evidenced by their lower degranulation ability and perforin expression (70, 71). Regarding the CD56^bright^ NK cell population, they express CCR7, a chemokine receptor associated with tissue trafficking; this has been noted to decrease. This decrease in CCR7 expression has been correlated with HIV viral load (72), supporting the idea that issues in NK cell trafficking contributes to a failure to contain HIV replication. Moreover, CD56^bright^ NK cells have also been found to be more expressive of TGF-β during HIV infection, which is of note as TGF-β inhibits immune effector functions (73, 74). As HIV infection progresses, an expansion in the amount of CD56^neg^ NK cells has also been observed (**Figure 2**) (71, 75, 76). In healthy subjects, the CD56^neg^ NK cell subset constitutes roughly 5.7% of NK cells in the peripheral blood, which has been shown in a meta-analysis to increase by an average of 10.6% after HIV-1 infection (77). CD56^neg^ NK cells have significantly impaired cytotoxic function due to expression of a relatively high quantity of inhibitory receptors (78). Furthermore, this cell population is known to produce a greater quantity of TGF-β, a cytokine associated with anti-inflammatory and homeostatic functions. TGF-β may also hinder autologous CD8 T cell functionality and potential to generate IFN-γ (79). The CD56^neg^ NK cell population is also known to expand in other chronic viral infections including hepatitis C virus (HCV), Epstein-Barr virus (EBV), and chronic cytomegalovirus (CMV). This observation suggests that it is likely a common feature among chronic viral infections (80).

During HIV infection, the expression of activating natural cytotoxicity receptors (NCRs) like NKp80 and NKp46 in NK cells are suppressed (81). Regarding inhibitory receptors, HIV viremia is associated with a significant increase in the expression of inhibitory KIR receptors, while NKG2A has been shown to decline, and taken together, the alterations in the expression of both activating and inhibitory receptors is associated with diminished cytolytic function (82). Additionally, the expression of CD69, CD18, and CD11b are also decreased, further suggesting functional impairment (83). A decrease in IFN-γ production by NK cells has also been observed, and coinfection with hepatitis C virus (HCV) is found to synergistically impair functional ability, with even further reduced IFN-γ and degranulation ability (84, 85).

To avoid targeting by cytotoxic T-lymphocytes, HIV selectively downregulates MHC-I molecules HLA-A and HLA-B, but largely retains HLA-E and HLA-C in some individuals in an attempt to avoid lysis by NK cells (86). This occurs in a mechanism dependent on HIV’s Nef protein and results in the aforementioned surface MHC-I molecules being rapidly internalized and degraded (87). Similarly, HLA-C has been shown to be downregulated by HIV-1’s viral protein U (Vpu) in some individuals, though not all (88, 89). HIV has been shown to induce the expression of ligands for NKG2D in infected cells by a mechanism dependent on its Vpr protein (90). The subsequent NK cell activation is speculated to contribute to CD4 T-cell depletion and NK cell dysfunction as sustained exposure to its ligand desensitizes the NKG2D receptor, resulting in its downregulation and diminished NK cell cytotoxic activity (91, 92). However, it is worth noting that NKG2D signaling alone is not enough to trigger NK cell degranulation as it requires coactivation with NK-T-B-antigen (NTB-A) which is downregulated through the action of Vpu enabling escape from the NK cell response (93). Also of note is that HLA-E is capable of displaying HIV antigen that blocks interactions with NKG2A limiting inhibitory NK cell signaling by this mechanism. This contrasts with HLA-C, which remains unblocked during HIV infection resulting in conserved inhibitory signaling via KIR receptors. This suggests that HIV^+^ cells are uniquely susceptible to NKG2A expressing NK cells that lack inhibitory KIR receptors (94), and therapeutics taking advantage of this possibility deserve further exploration. Similarly, the blocking of interactions between HLA-A and HLA-C with NK cell inhibitory receptors have enhanced cytotoxicity against HIV infected cells (95).

During early HIV infection, NK cell numbers and distribution remain relatively unaltered. KIR^+^ NK cells remain rare at this stage, and the relative naivety of the NK cell repertoire limits their ability to control the infection (96). As HIV infection progresses, there has been an observed increase in the number of CXCR5^+^ NK cells in lymphoid tissue (97). This population of NK cells highly express activating receptors, including NKG2D and NKp44, although they do demonstrate functional impairment, with reduced degranulation compared to CXCR5^+^ NK cells in non-infected individuals. Most notably, the frequency of these cells is negatively correlated with HIV-1 burden in the lymph tissue, illustrating their potential importance in controlling the HIV-1 reservoir.

Also found within lymph tissue is a subset of CD56^bright^ NK cells which express a high quantity of NKG2A inhibitory receptors, as well as NKG2D, NKp46, and NKp30 activating receptors (98). This subset is also relatively immature in regards to its differentiation status and cytotoxic potential due to expressing only low quantities of CD57 and CD16. In humanized MISTRG-6-15 mice, after HIV-1 infection, NK cells in the lymphoid tissue were relatively less mature and similarly more dysfunctional when compared to NK cells in the nonlymphoid tissues, consistent with this previous result (99). However, this observation may be in part driven by mismatched trafficking receptors impairing infiltration of NK cells into lymphoid tissue in this mouse model. Within the gut mucosa, two distinct subsets of NK cells located in the intraepithelial spaces and lamina propria are significantly diminished in prevalence during chronic HIV infection which contrasts with spontaneous HIV controllers who have a stable intraepithelial subset (100).

In terms of KIR receptor expression, the expression of the activating KIR2DS4 has been shown to decrease while the expression of other inhibitory KIRs remains constant with exception of KIR3DL2 which increases (101). Interestingly failure to downregulate KIR2DS4 has been associated with increased viral replication possibly due to promoting excessive NK cell activation in chronic HIV (102, 103). Similarly, the possession of inhibitory KIR receptor KIR2DL3 has been associated with increased chronic immune activation, especially when an individual is also a carrier of HLA-C2 (104). This same study also noted that carriers of activating KIR3DS1 also display heightened immune activation during chronic HIV. Further study has shown that KIR3DS1^+^ NK cells in the presence of the receptor’s cognate ligand display enhanced ability to control HIV infection *in vitro* and result in lower viral set points *in vivo* (105, 106). Overall, these studies again highlight the importance of an individual’s KIR and HLA repertoire, as the presence of certain receptor-ligand genes are associated with different functionality in HIV.

Mounting evidence also suggests that HIV infection is associated with an increase and persistence of memory NK cells (107). For example, macaques infected with either SIV or SHIV have been found to develop a subset of antigen specific NK cells present in the spleen and liver that specifically lyse dendritic cells pulsed with viral antigens like GAG or ENV (108). This mechanistically occurs in an NKG2C dependent manner and markedly contrasts with NK cells isolated from healthy macaques that lysed fewer cells in a non-specific manner. In humans, the presence of rare HIV peptide specific NK cells has been observed and is also associated with lower viral load, higher CD4 counts, and stronger HIV specific T-cell responses (109, 110). Further study is required to elucidate the mechanism by which these cells arise and to explore their function in greater detail. An expansion in NKG2C^+^ memory NK cells have also been described in early HIV infection and patients with high NKG2C^+^ CD57^+^ NK cell frequencies have been found to show lower immune activation and HIV RNA one month after starting antiretroviral therapy (ART) (111). The patients in this study were seropositive for cytomegalovirus (CMV), so from this data it is indeterminable the degree to which this memory NK cell population was induced by HIV infection itself. Regardless, further characterization of the functional role of NKG2C^+^ CD57^+^ memory NK cells in HIV is needed.

ART does not normalize the phenotypic changes observed in the CD56^dim^, CD56^negative^, and CD56^bright^ NK cell subsets caused by HIV infection (112). Deficiencies in the prevalence of CD56^dim^ and CD56^bright^, as well as the expression of CD107a, Granzyme-B, NKG2D, and NKp46, remain following ART therapy suggesting prolonged NK cell functional impairment (113). In spite of functional impairment, NK cell activation persists following viral suppression by ART (114). However, the cytokine-induced expression of IFN-γ and activating receptor NKp44 are also reduced, and CD56^bright^ cell counts are inversely correlated to the decreased T regulatory cell counts observed during ART, suggesting autoreactivity (115). In contrast, the relative expression of inhibitory receptors after ART does appear to return and is comparable to healthy donors (82). Despite ART, the dysregulation of NK cells in HIV is a clear component of the infection’s immunopathogenesis and is an avenue that many of the therapeutics discussed later are targeting for cure strategies.

### 2.2 Hepatitis C Virus (HCV)

Hepatitis C Virus (HCV) belongs to the Flaviviridae family and has a single-stranded RNA-genome (116). HCV primarily infects hepatocytes, but can also replicate in macrophages, B cells, and T cells, among other extrahepatic tissues (117). While HCV infection is often asymptomatic, chronic infection is frequently associated with the development of liver fibrosis and in severe cases cirrhosis (118).

**Figure 3:**
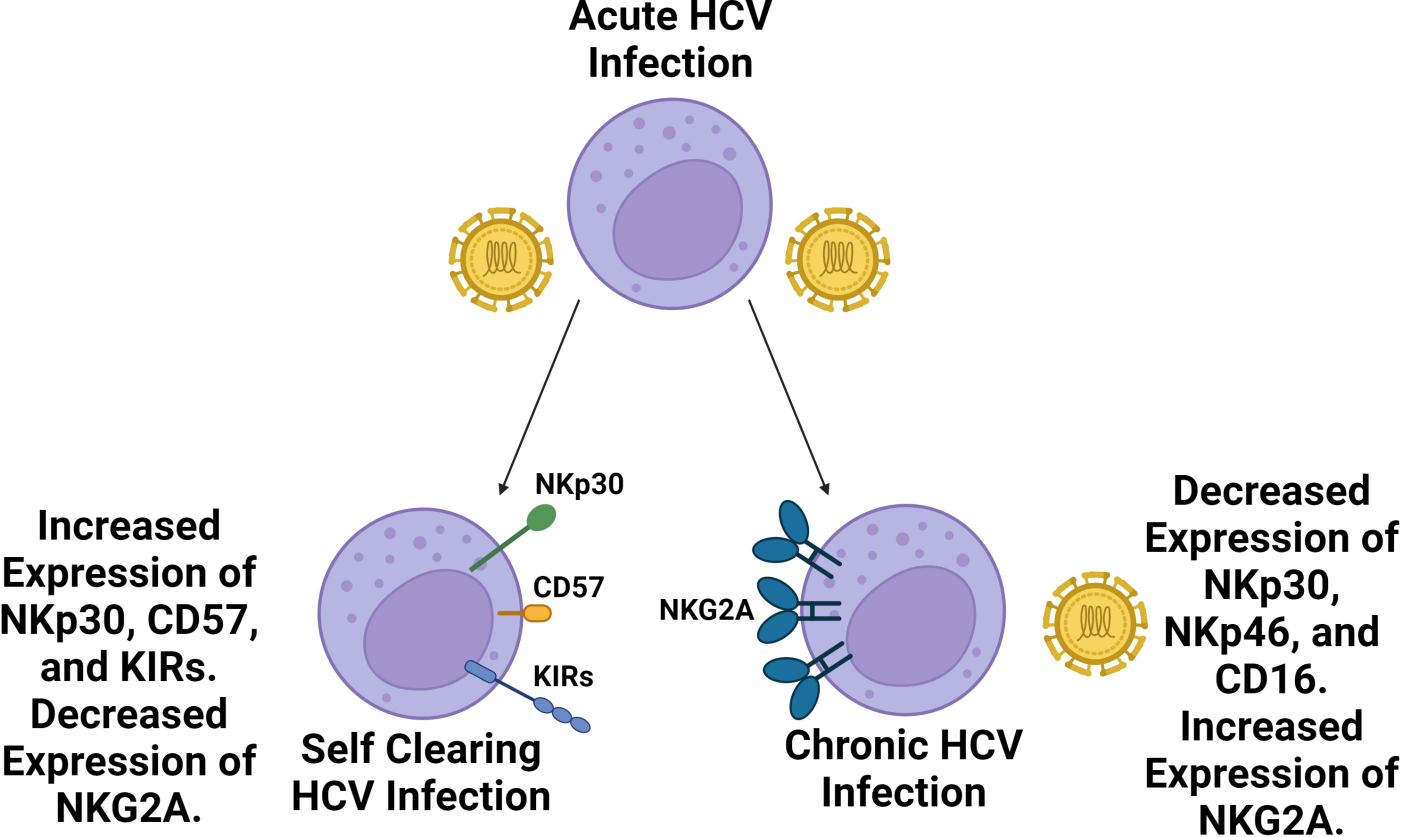
Self clearing HCV infections are associated with a more highly differentiated NK cell repertoire compared to chronic HCV infections. Following acute HCV infection, two outcomes may follow. In roughly a quarter of cases, HCV infection is spontaneously cleared whereas roughly three quarters of cases become chronic. Spontaneously cleared cases are associated with a more a more differentiated NK cell phenotype with increased prevalence of NKp30, CD57 and KIR expression as well as decreased expression of NKG2A. Chronic HCV cases are associated with diminished NK cell expression of NKp46, NKp30, and CD16 along with increased expression of NKG2A. NKG2A inhibitory signaling has been identified as a key source of NK cell exhaustion in HCV infection.

In regards to the NK cell response, the relative prevalence of CD56^bright^ NK cells increases, while the prevalence of CD56^dim^ NK cells slightly decreases in the blood during acute HCV infection (119). Additionally, both subsets have been found to upregulate the expression of activating receptor NKG2D while demonstrating strong cytokine production and cytotoxic activity. Following acute infection, approximately 25% of individuals spontaneously clear the virus, though the virus persists in the majority of individuals leading to chronic infection (120). Self-clearing HCV infections have been associated with highly differentiated NK cell phenotypes with increased expression of CD57 and KIR receptors (KIR2D, KIR3DL1, and KIR3DL2) and decreased expression of NKG2A (**Figure 3**) (121). Furthermore, certain NK cell phenotypes, like the expression of NKp30, are associated with spontaneous clearance (122). Homozygosity in KIR2DL3 and HLA-C1 has also been strongly associated with spontaneous clearance (43). The expression of inhibitory receptors NKG2A and inhibitory KIR2DL3 has been found to increase during chronic HCV infection while the expression of NKp30 and NKp46 was decreased relative to healthy controls (123, 124). Additionally, possession of HLA-B*44, HLA-C*12, and KIR3DS1 have been associated with failure to spontaneously clear HCV (125). Overall, the correlation of self clearance with the presence of mature, functionally active NK cells, as well as the presence or absence of specific KIR receptors suggests NK cell involvement in determining the chronicity of HCV.

Chronic HCV infection has also been associated with a significant decline in the total number of NK cells, specifically the CD56^dim^ subset in the peripheral blood (126, 127), similar to what occurs during chronic HIV infection. Additionally, the relative proportion of CD56^bright^ NK cells in the blood appears to increase during chronic infection due to both expansion of the subset, and the decline of CD56^dim^ NK cells in the blood (128, 129). For example, the CD56^dim^ NK cell subset has been observed to shrink to 4.9% of total lymphocytes in the blood during chronic HCV infection from 9.0% in healthy controls (123). In an *ex vivo* study, extracellular HCV-core protein was found to be correlated with the frequency of CD56^bright^ NK cells, suggesting that the protein attenuates NK cell differentiation and may help explain the increased prevalence of more naive CD56^bright^ NK cells (130). This direct viral modulation, along with other explanations providing rationale for the changes in NK cell subset prevalence in the blood during chronic HCV infection like tissue homing, would benefit from further investigation.

NK cells in the liver are predominantly of the CD56^bright^ subset, and liver fibrosis is associated with the diminished expression of activating receptors, including NKG2D and NKp46 (131). During chronic HCV infection, IL-26, has been found to be overexpressed (132). IL-26 is a cytokine that was recently identified as an inflammatory mediator, which binds extracellular DNA from damaged cells and induces activation of myeloid and lymphoid cells (133). The overexpression of IL-26 during HCV in turn upregulates TNF-related apoptosis-inducing ligand (TRAIL) expression on CD56^bright^ NK cells, increasing their ability to kill HCV-infected hepatoma cells (132). Additionally, an increase in the number of intrahepatic NKp44^+^ NK cells has been observed during chronic HCV (134). These intrahepatic NKp44^+^ NK cells are potent producers of TNF-α, and their frequency has been positively correlated with both HCV-RNA and fibrosis stage, indicating they may play a role in liver damage. It has also been observed that the relative prevalence of resident liver NK cells relative to circulating NK cells increases in the setting of cirrhosis, further suggesting NK cell involvement in HCV related liver fibrosis (135). Further work exploring if the NK cell response in the liver is mediating liver fibrosis or attempting to inhibit it through IL-10 and other mechanisms is needed to better understand the pathogenesis.

Functionally speaking, NK cells from HCV infected patients have a reduced capability to degranulate and produce cytokines (136). As infection progresses, reduced expression of NKG2D, NKp30, NKp46, and an increase in the CD56^neg^ NK cell subset and NKG2A expression is observed (137). Remaining CD56^dim^ NK cells also express CD16 to a lesser degree, limiting their ADCC ability (128). Additionally, hepatocytes in humanized mice upregulate Qa-1 (a murine functional homolog to HLA-E), which significantly contributes to NK cell exhaustion through inhibitory NKG2A receptor signaling (138). Furthermore, anti-NKG2A antibodies have been shown to restore NK cell production of IFN-γ in mice, resulting in decreased viral replication, demonstrating the relative importance of this pathway in NK cell exhaustion. Such immune checkpoint inhibitors will be explored further later.

Direct acting antiviral (DAA) therapy has been shown to normalize many of the changes in the NK cell repertoire associated with chronic HCV infection. For example, the prevalence of CD56^dim^ NK cells increases and the prevalence of the CD56^bright^ NK cell subset decreases, reverting both to levels comparable to healthy controls (139, 140). DAA has also been associated with decreased NK cell activation in the periphery, as well as decreased IL-12, IL-18, NKp30, NKp46, and TRAIL expression (139, 141). This further supports the idea that DAA, and by extension the decline in viral titers it causes results in diminished NK cell activation, and also teases NK cell involvement in the mediation of liver damage, due to treatment resulting in declining NK cell activation.

### 2.3 Cytomegalovirus (CMV)

**Figure 4:**
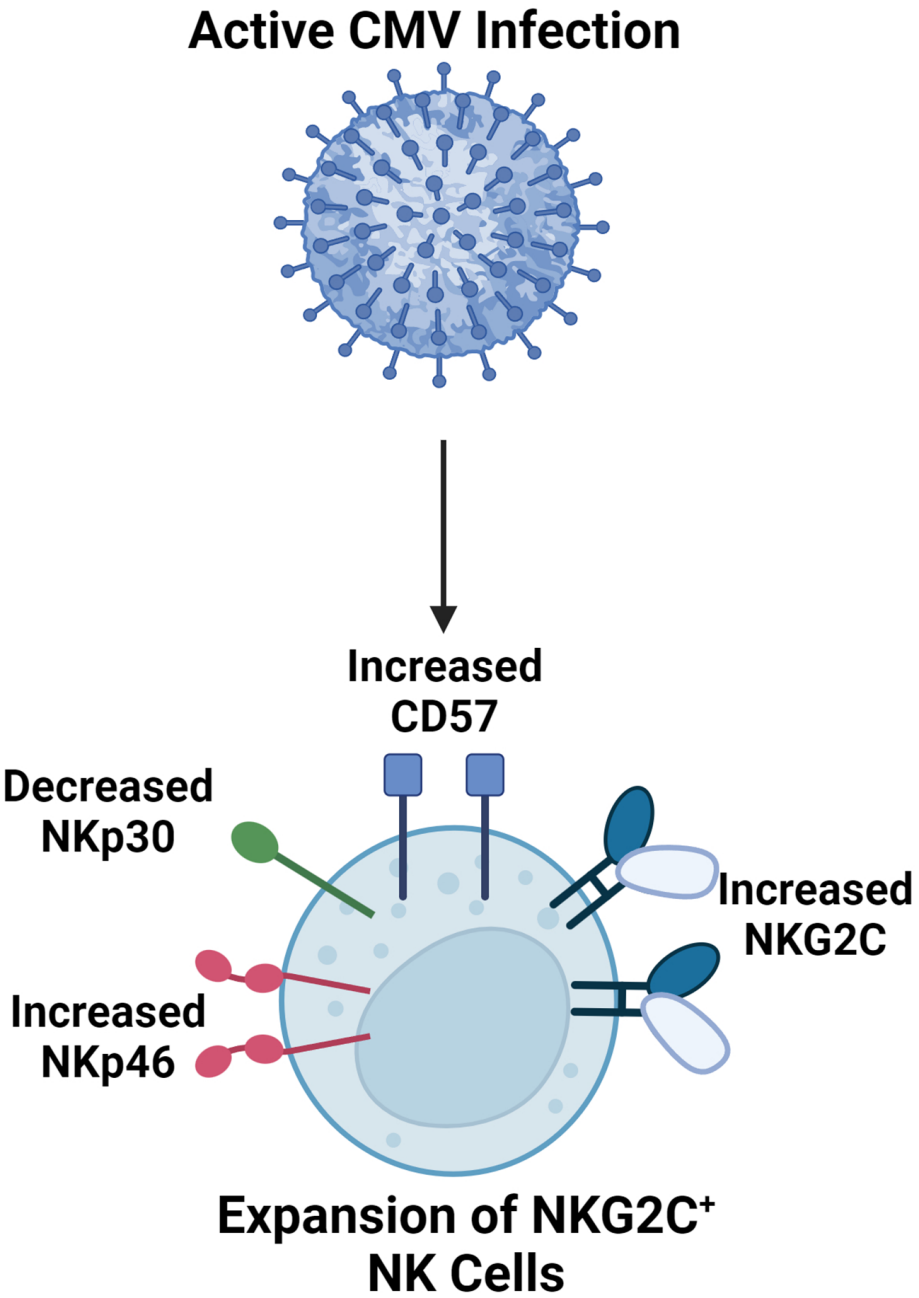
Acute CMV Infection and Subsequent Reactivation is Associated with an Expansion in NKG2C^+^ memory-like NK cells. These cells are phenotypically mature, long lasting, and exhibit enhanced functional potential leading them to be coined adaptive or memory like NK cells. This population is phenotypically defined by the expression of NKG2C and is associated with a high level of expression of CD57, NKp46, and inhibitory KIR2DL receptors.

Cytomegalovirus (CMV) is a member of the Herpesviridae family and has a large linear double-stranded genome (142). The virus infects a wide assortment of cells including epithelial cells, fibroblasts, monocytes, and macrophages and establishes a latent reservoir that is prone to later reactivation (143). The most notable feature of NK cells in CMV infection is the expansion of NKG2C^+^ CD57^+^ memory NK cells, also known as adaptive NK cells (144–147). These cells are phenotypically mature CD56^dim^ NK cells that express NKG2C, and persist after acute infection (148). Further, this NKG2C NK cell population preferentially expresses the inhibitory KIR2DL receptors, which has been described as contributing to increased functionality (149). CMV seropositivity is also associated with a decline in the expression of NKp30 and an increase in NKp46 (**Figure 4**) (150).

A similar expansion of NKG2C^+^ CD56^dim^ NK cells has been observed during CMV coinfection with viral hepatitis (both hepatitis B and C), suggesting that CMV-related NKG2C expansion is prominent even in the setting of other viral infections (151). This NKG2C^+^ memory NK cell population has been found to be have a greater capacity for ADCC and a potent capability to produce TNF-α and IFN-γ (152). As described earlier, these NKG2C^+^ CD57^+^ NK cells have been coined adaptive NK cells due to their selective proliferation, persistence, and functional potency in the setting of CMV infection, comparable to that of the adaptive immune system (153). The emergence and persistence of NKG2C^+^ CD57^+^ NK cells secondary to CMV infection are thought to be driven by epigenetic changes (154, 155).

Hematopoietic stem cell transplant (HSCT) recipients who experienced CMV reactivation post-transplant show a similar increase in the proportion of adaptive NK cells (156). These adaptive NK cells persist in the body for at least a year in post-HSCT recipients (157). In contrast, CMV seronegative recipients retain a population of comparatively less mature NK cells, presumably offering these recipients less protection. This is especially important considering that NK cells are among the first lymphocytes to recover status post-HSCT (158). This expansion of NK cells associated with CMV reactivation is also linked to a significant increase in the number of all NK cell subsets that persists even without further viral reactivation (157). Furthermore, NKG2C^+^ NK cells transplanted from CMV seropositive donors display heightened function during CMV reactivation (159). This supports the idea that NKG2C^+^ memory NK cells are also potentially transplantable and have rationale as a therapeutic which deserves further exploration.

### 2.4 Influenza Virus A (IVA)

Influenza A Viruses are members of the Orthomyxoviridae family and have a segmented genome consisting of 8 linear RNA strands (160). They are further subtyped based on the hemagglutinin (HA) and neuraminidase (NA) they express, which are surface glycoproteins that help mediate viral entry and exit respectively (161). Influenza viruses primarily infect epithelial cells of the upper and lower respiratory tract, and severe cases can result in acute respiratory distress syndrome (162).

Influenza virus A (IVA) infection induces the infiltration of NK cells into lung tissue with a significant increase in the number of NK cells expressing CXCR3, CXCR6, and CCR5, all of which are markers of tissue homing (163, 164). In mice, this infiltration is also in part regulated by IL-15 as evidenced by the blocking of IL-15 delaying this infiltration (165). Severe infection is associated with a decline in the total number of NK cells in the peripheral blood, particularly among CD56^bright^ NK cells, along with marked NK cell activation evidenced by an increase in the proportion of NK cells expressing CD69 (166, 167).

As a whole, there is a decrease in the number of NK cells expressing NKp46 but an increase in the number of cells expressing coreceptor 2B4 in the blood and these cells also show a greater degree of activation (168). Of the remaining NKp46 expressing NK cells, the proportion of NKp46^+^ NKp44^+^ NK cells expands while the proportion of NKp46^+^ NKp44^-^ declines, contributing to the shift towards NK cell activation (169). An NKp46^+^ NKG2A^+^ NK cell subset has also been found to be induced by IVA infection. This subset of NK cells has demonstrated a memory phenotype in that upon reencountering previously exposed influenza antigen, they display decreased cytotoxicity and increased IFN-γ production (170). Influenza is also directly capable of infecting NK cells, triggering apoptosis, and infection has been shown to inhibit NK cell cytotoxic function (171–173).

In the lung, tissue resident NK cells comprise 10-25% of the NK cell population meaning that most NK cells in the lung are circulating NK cells (15). The majority of lung tissue resident NK cells are CD16^-^ and have similarly been divided into CD56^dim^ and CD56^bright^ subsets in some studies (15). Functionally, the CD56^bright^ lung tissue resident NK cells subset has been described as showing greater functionality, with increased degranulation and IFN-γ production in response to IVA infection compared to other NK cells *ex vivo* (174). The rationale behind this subsets enhanced function is poorly understood and whether or not it translates *in vivo* is unknown.

**Figure 5:**
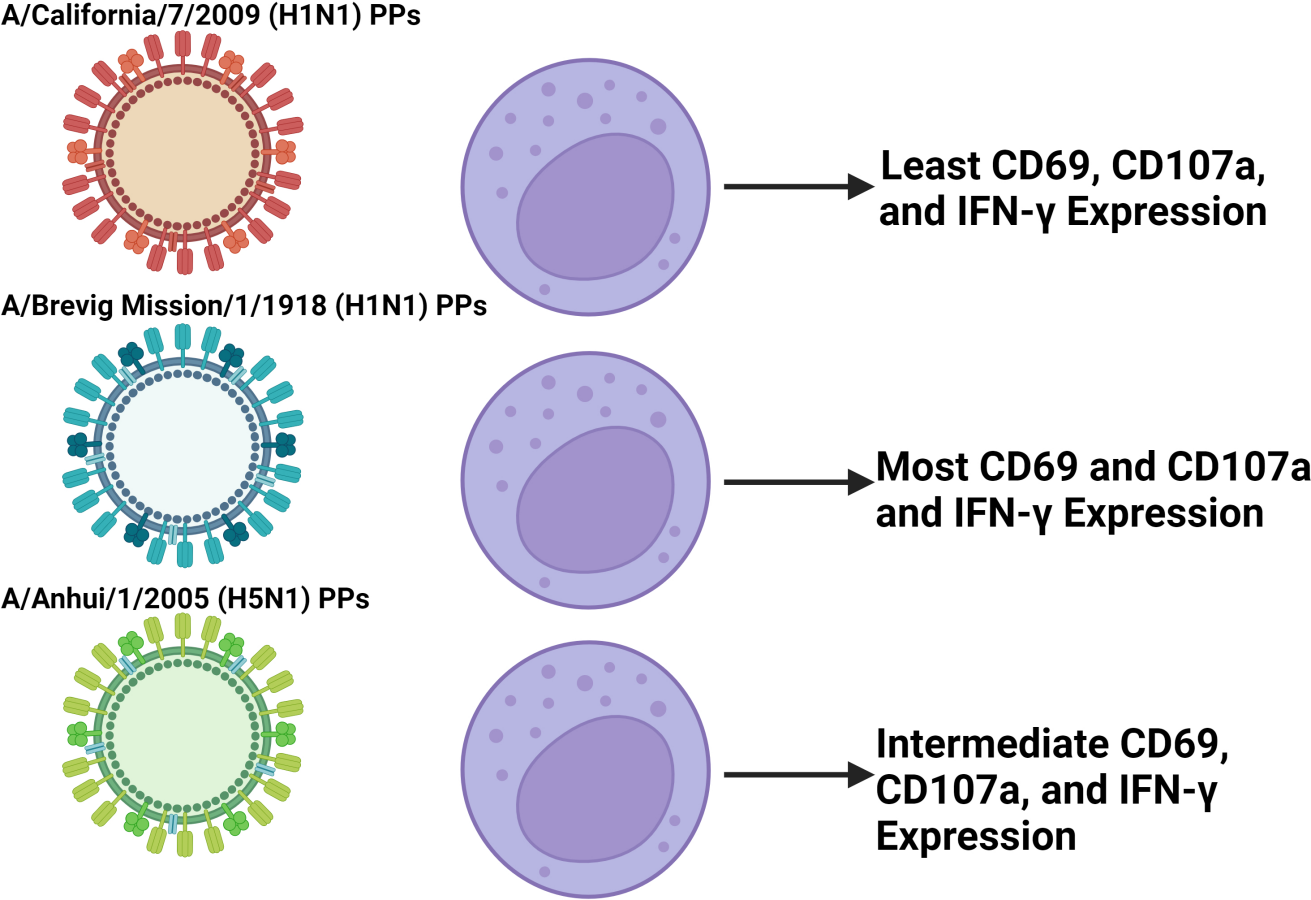
Coculture of NK cells with different IVA PPs induce differential NK cell activation. *In vitro* experiments coculturing NK cells isolated from PBMCs with pseudotype particles (PPs) of different IVA viruses found differential activation between PPs in terms of CD69, CD107a, and IFN-γ expression (139). The findings presented in the figure correspond to the 500 HAU/mL IVA coculture dose. The PPs modeling the more virulent 1918 H1N1 and H5N1 IVA viruses induced greater activation than the less virulent 2009 H1N1 PPs. This suggests that in IVA infection, disparities in early NK cell activation between influenza viruses may play a role in contributing to differential virulence between them. Further work is required to characterize this effect *in vivo* with whole virus that is not replication deficient due to IVA infecting and killing NK cells.

Differential activation of NK cells has been observed following coculture with replication deficient influenza A pseudotypes that express IVA HA and NA (175). Particularly, more robust upregulation of CD69, CD107a, and IFN-γ along with downregulation of NKp46 is observed with H5N1, and the 1918 H1N1 compared to 2009 H1N1 (**Figure 5**) (175). More broadly speaking, Influenza induces both degranulation and cytokine production, particularly in CD56^bright^ NK cells, and while their activation contributes to host immunity, it may well contribute to tissue damage as well (176). For example, NK cell-depleted mice infected with a high dose of influenza A virus have decreased mortality and disease morbidity illustrating how the NK cell response can be deleterious (177). Conversely, a study looking at NK cell responses in chickens challenged by either a weakly pathogenic H9N2 avian influenza virus or a highly pathogenic H5N1 avian influenza virus found that the less pathogenic avian virus induced a more vigorously activated NK cell response (178). This shows that a notable NK cell response in influenza is not always associated with viral virulence and may even be protective. Additionally, CD94 expression has been negatively correlated with influenza symptom severity, suggesting that signaling through CD94 containing receptors plays a role in control of IVA infection (179). Overall, more work is required to characterize differential NK cell responses between influenza viruses, and what correlates are predictive of deleterious responses.

The IFN-γ response orchestrated by NK cells is particularly important in the context of infection. Notably, impairment of NK cell activation and IFN-γ secretion is associated with weakened cytotoxic T lymphocyte activity against influenza (180). This has been corroborated in mice, and the initial increase in production of IFN-γ after NK cell activation results in downstream cytotoxic function in CD8 and NK cells during influenza infection (181). This potent IFN-γ response orchestrated by NK cells has also been implicated in contributing to thymic atrophy during influenza A infection, showing it can also be deleterious (182).

Influenza vaccination has also been shown to prime NK cell function with patients displaying increased production of IFN-γ and enhanced activation after stimulation, while NK cell phenotype and subset is largely unaltered (183, 184). However, influenza vaccination does induce a proportion of CD56^dim^ NK cells to become memory NK cells and express intracellular NKp46, offering a possible mechanism by which the IFN-γ NK cell response is enhanced after vaccination (185). These vaccine induced memory NK cells display a heightened IFN-γ response that persists 6 months after vaccination. It has also been found that NK cells in mice vaccinated with a multivalent influenza vaccine had greater expression of granzyme B and perforin and took on a more mature phenotype after being challenged by heterosubtypic influenza strains compared to unvaccinated mice (186). This again supports the notion that influenza vaccination primes NK cells to offer greater protection even against heterosubtypic strains where vaccine-derived humoral immunity may struggle. Changes in the NK cell population induced by other vaccines remain underexplored, and examining these NK cell phenotypic changes induced by vaccines as a correlate of immunity warrants more attention.

### 2.5 SARS-CoV-2

SARS-CoV-2 belongs to the Coronaviridae family and has a large single-stranded RNA genome (187). The virus primarily infects ACE2^+^ cells of the respiratory tract, particularly type II alveolar pneumocytes and in severe cases can result in acute respiratory distress syndrome (188). Severe COVID-19 disease has been linked to a significant decline in CD56^+^ CD16^+^ NK cells in the blood (189–191). This decrease in circulating NK cells is correlated with acute SARS-CoV-2 infection and is associated with disease severity (192, 193). In hospitalized patients, NK cell counts correlate directly with the speed of viral load decline. For example, those with normal NK cell numbers in the blood showed a faster decline of viral load compared to those with low NK cell numbers, suggesting that circulating NK cells may represent a prognostic clinical marker of COVID-19 severity and outcome in adult patients (194). When patients recover after COVID-19, their circulating NK cell counts return to normal. In patients affected by long COVID-19, NK cell counts have recovered, despite continued symptoms (195). In Multisystem Inflammatory Syndrome in Children (MIS-C), a severe post-infectious complication that occurs 4-6 weeks after COVID-19 infection, CD56^dim^ NK cells are also decreased in number (196).

During COVID-19, NK cells are robustly activated, evidenced by an increase in the expression of CD69, and KI-67 a proliferation marker (192). Expression of the genes KLRD1 and KLRG1, which encode CD94 and killer cell lectin-like receptor G1 respectively, are also noted to decrease in many patients contributing towards NK cell activation (197).

SARS-CoV-2 infection has also been linked to an increase in the expression of NKG2C, creating a population similar to the earlier described memory NK cells (192, 198). Interestingly in patients with adaptive NK cells, there was no detectable circulating CMV DNA, suggesting that the expansion of adaptive NK cells in severe COVID-19 patients may be independent of CMV reactivation secondary to COVID-19. It is still to be determined if SARS-CoV-2 itself can drive the expansion of adaptive NK cells or if they are driven by the production of pro-inflammatory cytokines. In those affected by long COVID-19, there are increased levels of CD57^+^ NKG2C^+^ memory NK cells; these are one of the variables important for predicting a higher susceptibility to develop long COVID-19. However, despite greater populations of memory-like NK cells, their effector functions remained impaired (195).

The expression of cytotoxic effector molecules like perforin and Granzyme A are also significantly increased during most COVID-19 cases (189, 199, 200). While these cytotoxic effectors are upregulated, NK cells from COVID-19 patients have been shown to have reduced degranulation potential and cytotoxicity (201). Furthermore, in severe cases, the quantity of TNF-α and IFN-*γ* produced by NK cells is diminished (202–204). Inhibitory KIR and NKG2A receptors have been found to be upregulated during COVID-19 and likely contribute to NK cell exhaustion (205, 206) NKG2A is also highly upregulated in acute, severe COVID-19 patients, while mild and moderate patients show a recovery of NKG2A after resolution of the infection (198). Significant NK cell exhaustion is further supported by the increased frequency of programmed cell death protein 1 (PD-1), Lymphocyte-Activation Gene 3 (LAG-3), and TIGIT, as well as a decreased frequency of NKG2D. However, the level of PD-1 expression in NK cells isn’t correlated with disease severity (203, 204), while decreased expression of TIGIT and DNAM-1 have been associated with slower viral clearance (207). Overall, more work is needed to ascertain the degree to which exhaustion is responsible for NK cell’s diminished function in COVID-19, as opposed to issues in tissue homing.

**Figure 6:**
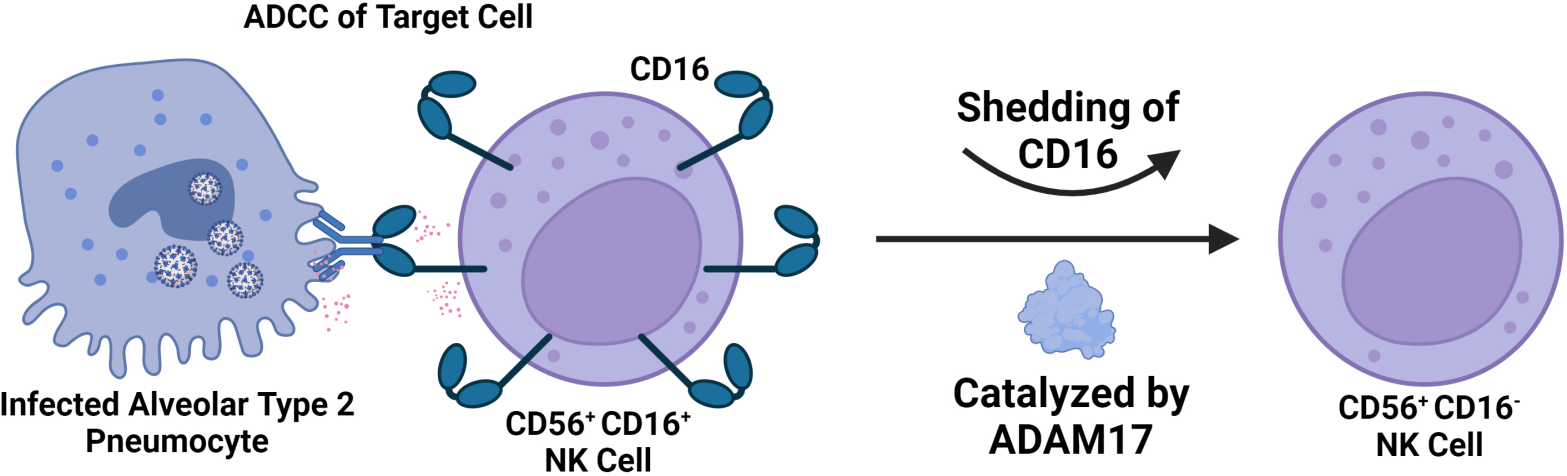
Severe SARS-CoV-2 is Associated with a Significant decrease in the number of CD56^+^ CD16^+^ NK Cells in the Blood. The decline in CD56^+^ CD16^+^ NK Cells is due to CD16 shedding catalyzed by ADAM17. This results in an accumulation of CD56^+^ CD16^-^ NK cells of less cytotoxic potential and in severe cases likely contributes to impaired capability to engage in ADCC. CD16 mediates ADCC by binding to antibodies attached to target cells, ultimately resulting in degranulation.

Infection has also been linked to the expansion of the CD56^dim^ CD16^neg^ NK cell population, which has decreased cytotoxic potential compared to the standard CD56^dim^ CD16^+^ NK cell subtype (**Figure 6**). It has also been shown that CD56^dim^ CD16^neg^ NK cells expand in the early phases of SARS-CoV-2 and then decrease in mild or moderate cases, but in severe COVID-19 cases, this subset continues to expand (208). The study also suggests that loss of CD16 is potentially being mediated by cleavage through Adam17 in these cells (208). In MIS-C, studies have shown that there is a decrease in the prevalence of CD16^+^ NK cells, suggesting that there is a decrease in NK cell ADCC function in MIS-C (200). However, further studies are needed to explore NK cell function in MIS-C directly beyond just immunophenotyping.

In fatal COVID-19 cases, patients had impaired upregulation of perforin, granzyme-A, and KI-67, suggesting that defects in NK cell cytotoxic activity are associated with increased morbidity and mortality (209). Corroborating this is the observation that NK cell mediated ADCC appears to be impaired in severe COVID-19 (210). In the context of COVID-19 infection, TGF-β signaling has been associated with a stark reduction in NK cell effector functions, with patient serum having been found to inhibit NK cell cytotoxicity in a TGF-β dependent manner (194). Similarly, impairments in IFN-*γ* production appear to occur in a TGF-β dependent manner (211). This suggests that TGF-β signaling contributes significantly to NK cell dysregulation and exhaustion during COVID-19.

## 3 NK cell therapeutics

Given the extensive changes in NK cell phenotype and repertoire, which often lead to exhaustion and dysregulated NK responses in viral infections, several therapeutic approaches with the goal of promoting NK cell function are under investigation. The majority of NK cell-related therapies originate from the domain of cancer biology and have only recently been studied in the context of viral diseases.

### 3.1 TGF-β inhibitors

The development of therapeutic TGF-β inhibitors largely stems from the implication of aberrant TGF-β signaling in the pathogenesis of cancer, cardiovascular disease, and fibrosis. As a therapeutic class, they span a wide variety of structures from small effector drugs to blocking antibodies targeting a diverse host of TGF-β pathway components (212, 213). TGF-β signaling in HIV infection has been documented to promote latency by upregulating the transcriptional repressor Blimp-1 (214, 215). Galunisertib, a TGF-β type I receptor inhibitor, was administered to SIV infected rhesus macaques, where it was shown to increase reactivation of the SIV latent reservoir, and enhance the subsequent immune response to SIV (216). TGF-β signaling during SARS-CoV-2 infection has also been associated with NK cell dysregulation that limits functional activity contributing to subsequent viral pathogenesis (194, 217), showing that TGF-β inhibitors may have rationale to be applied in acute viral infections too.

Broadly speaking, TGF-β signaling has been shown to impair NK cell cytotoxic function in part by restricting the IL-15 activation of the mTOR pathway (218). Additionally, deletion of the TGF-β receptor subunit TGF-βRII has been shown to promote the cytotoxic function of NK cells in response to IL-15, enhancing the ability of NK cells to limit metastasis in two different murine tumor models (218). It is worth noting that by inhibiting NK cell cytotoxic function and the secretion of proinflammatory cytokines like IFN-*γ*, TGF-β plays an important role in limiting excessive NK cell activation. The risk of NK cell hyperresponsiveness, and the subsequent inflammatory environment and tissue damage this induces, should be considered in the testing of TGF-β Inhibitors. However, considering the clear contribution of TGF-β signaling to NK cell dysregulation and evidence that restraining this signaling may actually promote NK cell cytotoxicity beneficially, there warrants further investigation into the use of TGF-β inhibitors as a potential therapeutic for treating viral infections.

### 3.2 Immune checkpoint inhibitors

A key component of NK cell dysregulation is exhaustion driven by inhibitory pathways. These inhibitory pathways have been termed immune checkpoints, and immune checkpoint inhibitors have been developed to interfere with these pathways in an effort to preserve immune function (219).

NKG2A is an important inhibitory receptor expressed on most NK cells and is considered an immune checkpoint for NK cells. NKG2A inhibitors like the monoclonal antibody monalizumab have been found to promote antitumor immunity in treating squamous cell carcinoma by enhancing the activity of NK and CD8 cells (220). Monalizumab has also demonstrated a similar response in the setting of murine colorectal cancer (221), and against HLA-E^+^ leukemia and lymphoma in mice (222). NKG2A blocking monoclonal antibodies have also shown promise in treating viral hepatitis, with cultured NK cells from patient’s demonstrating enhanced cytotoxicity (223). Similarly, NKG2A-blocking monoclonal antibodies have been shown to restore the production of IFN-γ by NK cells during HCV infection, and has been associated with diminished CD8 T cell exhaustion (138). This therapeutic also has rationale to be applied in treating COVID-19 due to NK and CD8 T cell exhaustion being driven in part by increased NKG2A expression (205, 224, 225).

Antibodies targeting inhibitory KIR receptors have also emerged as another NK cell related immune checkpoint therapy. One such KIR inhibitor named IPH2101 which targets inhibitory KIRs (KIR2DL-1, KIR2DL-2, and KIRT2Dl-3) recently underwent a phase 1 study where it was deemed safe and tolerable and in *ex vivo* assays demonstrated increased NK cell cytotoxicity against multiple myeloma (226). However, a phase 2 study of IPH2101 was terminated due to lack of clinical efficacy (227), and similarly, a trial for lirulumab, another anti-KIR antibody (which targets KIR2DL-1, KIR2DL-2, and KIRT2Dl-3) was terminated for failing to meet objective response criteria (228). IPH2101 has also be combined with the anti-tumor drug lenalidomide in further testing against multiple myeloma with early data suggesting a promising synergistic effect, although more study is required (229, 230). As this study highlighted, a key advantage of immune checkpoint inhibitors is that they can readily be combined with other therapeutics. IPH2101 has also been tested in the setting of acute myeloid leukemia, where a degree of early TNF-α and CD69 upregulation was detected (231). Another anti-KIR antibody named IPH4102, targeting KIR3DL2, has also been tested in relapsed and refractory cutaneous T-cell lymphoma, which showed its potential to elicit a promising clinical response in 36.4% of the study participants (232). Little testing of KIR inhibitors has been undertaken in the setting of viral infections, although they warrant study particularly in HIV due to HIV viremia being associated with an increase in the expression of inhibitory KIR receptors (82).

PD-1 signaling has been implicated as a hallmark of T-cell exhaustion and a contributing factor of immune escape in the setting of cancer, and is considered another important immune checkpoint (233, 234). PD-1 signaling has been shown to be of significance to NK cells in multiple myeloma (235), renal cell carcinoma (236), and in Kaposi sarcoma (237), where it contributes to an exhausted NK cell response (29). Several PD-1 checkpoint inhibitors have been approved by the FDA including pembrolizumab, nivolumab, and cemiplimab among many others, which together have been tested clinically in a diverse arrangement of cancers (238). Significant expression of PD-1 has been shown in chronic HIV (239), HCB (240), HCV (241), Influenza (242), and SARS-CoV-2 (243), with a similar exhaustive effect.

In a mice influenza challenge model, PD-1 inhibitors were shown to enhance CD8 T-cell function resulting in quicker viral clearance (244). In SIV infected rhesus macaques, the blockade of PD-1 was well tolerated and resulted in the expansion of functional SIV-specific CD8 T-cells in the blood and gut as well as the expansion of memory B-cells all of which was associated with lower viral load and prolonged survival (245). Similarly, in CD4^+^ humanized mice, PD-1 blockade was associated with lower HIV viral load and increased T-cell counts (246). In HBV, PD-1 blockade has also been shown to again promote CD8^+^ T-cell expansion and secretion of IFN-γ and IL-2 (247). Importantly, the upregulation of PD-1 in NK cells during acute febrile malaria has been associated with enhanced ADCC, but otherwise diminished degranulation (248). This finding hints that the impact of PD-1 blockade on NK cells, may be more nuanced in terms of impact on functional activity than CD8 T cell function. Overall, more work is needed to characterize the impact that PD-1 blockade has on the NK cell population in the setting of these viral infections; however it is clear based on the CD8 T-cell response alone that PD-1 blockade is a promising therapeutic that deserves further examination in the setting of infectious diseases (249).

### 3.3 Adoptive NK cell therapies

Another therapeutic approach entails the administration of exogenous NK cells. The goal is to supply functionally intact cells to supplement NK cell functional activity during virally mediated NK cell dysfunction and exhaustion. The administration of allogenic NK cells has been safely and effectively used to treat leukemia (250). Delivery of exogenous NK cells have also demonstrated clinical responses in treating non-small cell lung cancer (251), platinum-resistant ovarian cancer (252), melanoma (253), and renal cell carcinoma (253). Very little work has been done studying adoptive NK cell therapies in infectious diseases. In SARS-CoV-2 and HIV-1, an additional consideration is the infusion of adaptive NK cells due to their enhanced cytotoxicity and persistence (198).

Recently, genetically engineered NK cell lines derived from induced pluripotent stem cells (iPSC) have emerged. Some of the gene edits in these cells include a non-cleavable CD16a receptor, a membrane-bound IL-15/IL-15R fusion protein, and knockout of coenzyme CD38 to promote the persistence and function of these cells after administration (254). In the setting of HIV, allogenic NK cells derived from human embryonic stem cells have been shown to decrease HIV replication during acute infection in humanized mice, and when combined with latency reversal, helped diminish the HIV reservoir (255, 256). Furthermore, phase 1 safety trials where haploidentical NK cells are infused into HIV infected ART suppressed patients in combination with either IL-2 or N-803 (an IL-15 superagonist) supportive therapy have been completed (NCT03346499, NCT03899480).

There are clinical trials using primary and iPSC-derived or “off-the-shelf” NK cells as therapeutics against SARS-CoV-2, suggesting that adoptive NK cells transfer may be a viable therapeutic in the setting of acute viral infection. An example of this is a phase 1 study in which a line of IPSC derived NK cells called FT516 administered to hypoxic COVID-19 patients to assess their safety and maximum tolerated dose. Results of this study (NCT04363346) are anticipated soon. Taken together, the demonstrated safety of utilizing exogenous NK cells, along with preliminary findings suggesting possible efficacy in treating viral infections, particularly in HIV, calls for further investigation.

### 3.4 Cytokine stimulation and cytokine induced memory like NK cells

Approaches to enhance NK cell anti-viral functions *in vivo* and prior to adoptive transfer of exogenous NK cells are of interest. In oncology, many cytokines have been used for the treatment of NK cell dysfunction, such as IL-12, IL-15, and IL-18 given their ability to promote NK cell expansion and maturation (257–259). In the context of SARS-CoV-2, a phase 2 clinical trial has been completed looking at the efficacy of low-dose IL-2 in acute respiratory distress syndrome (ARDS) related to COVID-19 (NCT04357444). NK cells treated with IL-15 and stimulated with an inactivated whole influenza virus were shown to mount a more vigorous NK cell response compared to when not treated with IL-15 (260). In HIV, NK cells stimulated with IL-15 demonstrated uniformly improved effector functions and the ability to kill HIV-infected cells that were treated with a latency reversal drug (261).

IL-12, IL-15 IL-18 are also of particular interest given their ability to induce changes in NK cells through transcriptional, epigenetic, and metabolic mechanisms that ultimately increase their cytotoxicity and result in memory like behavior (262). Cytokine induced memory like (CIML) NK cells have displayed increased IFN-γ responses in addition to enhanced recall responses even after multiple rounds of cell division (263, 264). They also display increased expression of NKG2C, NKG2A, CD69, and CD94, but not CD57 or KIR receptors (263). Due to the enhanced effector functions and relative ease at which CIML NK cells are generated, extensive study of the adoptive transfer of these cells, especially in the setting of myeloid malignancies, multiple myeloma, and head and neck squamous cell carcinoma has been undertaken (262). For example, infusion of 5 to 10 million cells/kg in patients with myeloid malignancies on lymphocyte depleting chemotherapy displayed a 10 to 50 fold expansion of NK cells that was determined to be independent of CMV reactivation and persisted for months (265). Other phase 1 studies of adoptively transferred CIML NK cells has also documented clinical responses. In the setting of acute myeloid leukemia, one study found clinical responses in 5 of 9 patients, of which included 4 complete remissions (259). Another study found complete remission in 4 of 8 patients (266), and when supported with 3 weeks of N-803 an 87% complete response rate was achieved by day 28 in a different study (267). The adoptive transfer of CIML NK cells has been understudied as a possible treatment for viral infections, and preclinical work, especially in the setting of HIV should be prioritized.

### 3.5 IL-15 superagonist therapy

Due to the aforementioned benefit of IL-15 stimulation on NK cell effector functions, novel approaches to enhance IL-15 signaling *in vivo* have been investigated. One such therapeutic is the IL-15 superagonist ALT-803. In SIV-positive rhesus macaques, ALT-803 (now N-803), an IL-15 superagonist comprised of an N72D mutant IL-15 molecule attached to its alpha receptor and a human IgG1 fragment designed to increase IL-15 activity, was shown to increase the NK cell population in the peripheral blood and decreased viral loads (268). In SHIV-infected rhesus macaques, N-803 was shown to mediate migration of virus-specific CD8+ T and NK cells to B-cell follicles, however no latency reversal was observed (269). A recent phase-1 clinical trial of N-803, showed that this molecule is also safe and tolerable in people living with HIV that are ART-suppressed. Further, N-803 was associated with CD8^+^ T and NK cell activation, as well as induced HIV RNA expression, with concurrent reduction in HIV DNA in Lymphoid tissue over time (270). Future clinical trials are needed to test the effects of these molecules on HIV reservoirs due to the promising results of the aforementioned preclinical and phase 1 studies.

### 3.6 NK cell engagers

As mentioned above, viral infections, especially HIV, can deter the natural recognition of virally infected cells by NK cells due to the manipulation of activating and inhibitory receptors expressed by the infected cell, thereby evading the immune response (82, 86, 87, 90–95). Broadly neutralizing antibodies (bnAbs) have been identified to play a role in viral control (271), and are used as a treatment and prevention strategy (272, 273).

Furthermore, in the RV144 HIV vaccine trial, which showed a statistically significant decrease in HIV infection risk (274), one correlate of reduced risk was found to be increased IgG antibodies targeting the V1V2 loop of the HIV ENV protein (275, 276). In a follow-up analysis, it was shown that these IgG antibodies elicited Fc-mediated NK cell ADCC functions (277, 278). These data suggest that non-neutralizing antibodies play a role in HIV protection through mediating effective Fc-effector functions (279–281). Additionally, NK cell-mediated ADCC against HIV has been noted as an important mechanism for HIV control (282–284), despite NK cell dysfunction (75, 113). However, a major concern with HIV-specific bnAbs is that HIV mutates rapidly and can escape bnAb-mediated neutralization (285–288). Further, the majority of bnAbs, which are generally of the IgG1 isotype, have good neutralization capacity, but subpar Fc-effector functions compared to their IgG3 isotypes (289–291).

Engineered small molecules are a promising strategy to direct NK cell mediated killing. The cancer field has made great progress in this field with the development of NK cell engagers (NKCE), Bi- and Tri– specific killer engagers (BiKEs and TriKEs™) as well as multi-specific antibody-based constructs (ANKET and StitchMabs) (292–297), which bind to activating receptors on NK cells. Briefly, TriKEs™ consist of an anti-CD16 engaging molecule that directly binds the CD16 receptor, joined via a flexible linker to an IL-15 molecule, and in-turn joined via linkers to an antigen engaging molecule to target an antigen of interest (298). Similarly, BiKEs consist of a similar anti-CD16 engaging molecule that is joined via a linker to an analogous antigen engaging molecule (298). ANKET constructs consist of an Fab that targets an NK cell receptor, commonly NKp46, together with another Fab that targets an antigen of interest (292). As alluded to above, ANKET molecules can also be generated to display bivalent binding to one or two different antigens (292). Furthermore, T-cell based nano engagers have also been developed in the form of Bi-specific T cell engagers (BiTEs) (299), and dual affinity retargeting antibodies (DARTs) (300, 301), although this is outside the scope of this review and won’t be discussed further here.

If we consider these therapies for HIV infection, many of the NK cell engagers mentioned above use either NKp46 or CD16 as the NK cell engaging molecule. However, NK cells are dysregulated during HIV infection, with activating and inhibitory receptor expression modulated. Importantly, despite NK cell dysfunction during HIV infection, NK cells still retain expression of CD16, enabling them to mediate ADCC (82). Therefore, given NK dysfunction, the retention of CD16 and the importance of Fc-mediated effector functions to combat HIV, strategies that exploit these are promising. Engineered small molecules that can bind directly to NK cells via CD16 interaction have been recently developed (302). This has been achieved by utilizing the BiKE and TriKE™ constructs that had previously been designed to target cancers (303–308).

These molecules allow the targeting of a variety of antigens for multiple viral infections, while also mitigating potential issues that arise with Fc-mediated effector functions. Further the IL-15 molecule induces persistence, expansion, and activation of NK cells (257). Currently, a BiKE construct has been generated using the HIV binding region of the VRC01 bnAb. This VRC01 BiKE construct, was shown to bind the HIV infected cell lines HIV-IIIB and ACH-2, but not their uninfected counterparts H9 or CEMs. Further, this VRC01 BiKE mediated effective NK activation, assessed by degranulation via CD107a expression and IFN-γ and TNF-α cytokine production, and subsequent killing against these HIV infected cells (309). Further work generating and testing TriKE constructs making use of the VRC01 binding region as well as other known HIV bnAb binding regions is currently underway.

However, HIV can escape viral restriction via bnAbs, so breadth is an important consideration in the development of novel therapeutics. Importantly, all HIV variants must utilize the CD4 receptor for initial gp120 binding to the cell surface (310). Therefore, using the CD4 receptor as the HIV binding region in novel therapeutics could overcome HIV escape (285). However, as this is a naturally found receptor used by many cells, careful consideration must be taken in the design so not to induce self-targeting. Extensive work in this field has already been done (311–313), and a CD4 extracellular domain 1 (CD4ECD1) molecule, designated mD1.22, with potent HIV neutralization and little binding to MHC class 2 has been identified (314). When designed into a BiKE construct with CD16 binding capabilities, it induced NK cell degranulation and killed HIV target cells (315). A CD4ECD1 TRiKE is currently in development and testing. Further, these TriKEs can be used in combination with a checkpoint inhibitor therapy (308), or other cytokine based or adoptive NK transfer strategies, highlighting the vast potential of these NK cell engagers for combination therapies to combat viral infections.

### 3.7 CAR NK cells

Chimeric antigen receptor (CAR) cells are another promising treatment that have been popular in the cancer field for many years and are now starting to be studied as a potential therapeutic for viral diseases. Many researchers have begun to produce CAR NK cells as they are less expensive and less likely to cause cytokine storm than CAR T cells (316). One such example is a CAR-NK cell therapy that secretes IL-15 and expresses a CAR with an extracellular ACE2 domain to target the SARS-CoV-2 spike protein and has been promising against SARS-CoV-2 *in vitro* (317). There is also currently another clinical trial underway to test a CAR NK cell that expresses ACE2 on it surface to treat severe COVID-19 patients (NCT04324996).

**Figure 7:**
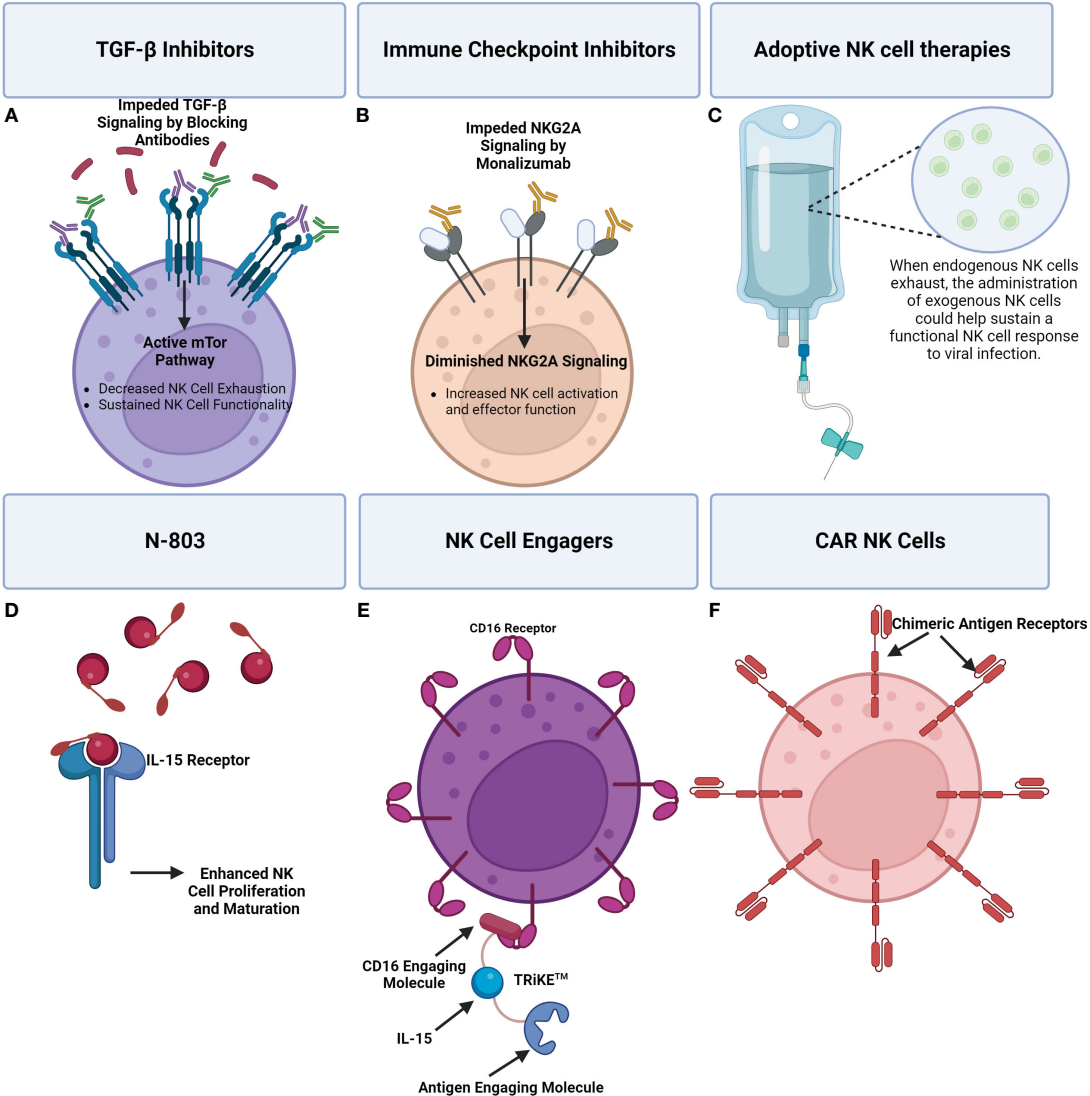
A visual overview of a few emerging therapeutics seeking to address NK cell dysregulation during viral infection. **(A)** TGF-β signaling inhibits the mTOR pathway which has been shown to result in impaired NK cell effector functions. The use of TGF-β inhibitors can promote mTOR signaling and in turn NK cell activity. **(B)** Immune checkpoint inhibitors can be applied to reduce inhibitory signaling that is causing NK cell exhaustion. For example, Monalizumab, an NKG2A inhibitor, can block inhibitory NKG2A contributing to a shift towards NK cell activation and effector functions. **(C)** Adoptive NK cell therapies entail the administration of exogenous NK cells to supplement the immune response. **(D)** N-803 is an IL-15 superagonist consisting of a mutant IL-15 molecule associated with an IL-15 receptor fusion protein. Administration of N-803 promotes NK cell proliferation and maturation. **(E)** An example of an NK cell engager are TRiKE™ constructs which consist of a CD16 engaging molecule linked to an IL-15 molecule that’s linked to an antigen linking molecule. Binding of the TRiKE™ to an NK cell CD16 molecule induces activation while IL-15 supports proliferation and maturation. The antigen linking molecule is designed to target an antigen of interest. **(F)** The extracellular antigen recognition domain of CAR receptors enables CAR NK cells to specifically target cells producing an antigen of interest. CAR NK cells can be administered to supplement an exhausted NK cell response, with less risk than CAR T-cells.

CAR NK cells are also currently being used in a clinical trial to treat HIV (318). In the setting of HIV, CAR-NK cells have largely targeted a single epitope of the HIV envelope glycoprotein gp160. Recently, the development of universal CAR-NK cells that target a broader range of gp160 has shown greater activation and the ability to kill HIV infected human CD4^+^ T cells (319). A visual overview of the discussed therapeutics is available in **Figure 7**.

## 4 Perspective on the use of NK cell therapies during viral infection

As described above, several lines of therapeutics including TGF- β inhibitors, adoptive cell therapies, cytokine therapies, NK cell engagers, and CAR NK cells have emerged in an effort to remedy NK cell dysregulation. While many of these therapeutics have been primarily tested in the setting cancer, they deserve further attention as possible remedies to preserve and restore NK cells function in the setting of viral infection. Furthermore, testing of these therapies in conjunction with each other as well as other therapeutics may prove particularly beneficial due to possible synergism.

Due to the extensive genetic differences present in NK cell receptors like the KIR family and ligands like HLA (31–33, 37), it is likely that there is also significant variation in the efficacy of these therapeutics depending on an individual’s personal repertoire of receptor and ligand genes. One of the clearest examples of this can be found in the case of KIR checkpoint inhibitors like IPH4102. Due to extensive variability in individuals’ KIR repertoire, responses to therapy between patients are also very variable, with only a subset achieving clinical responses (232). Similar variability in responses should be anticipated for the other therapeutics discussed here due to genetic variability in other NK cell receptors and ligands. Another consideration is that many of these therapeutics also run the risk of contributing to tissue damage and hyperinflammatory states by enhancing NK cell cytotoxic action and secretion of proinflammatory cytokines. Nonetheless, there may prove to be a balance between this risk and possible therapeutic benefit.

## 5 Concluding remarks

While NK cells play an important role in responding early to viral challenges, they can be dysregulated. This dysregulation, whether it be related to alterations in the prevalence of activating or inhibitory receptors, relative maturation in terms of subset prevalence, expression of functional effector proteins, or a product of an individual’s receptor and HLA genetics limits their ability to mount an effective antiviral response. For example, during chronic HIV and HCV, as well as acute SARS-CoV-2 there is an inherent depletion in the total prevalence of CD56^+^ CD16^+^ NK cells in the peripheral blood which is accompanied by markedly increased NK cell exhaustion. In contrast, aberrant NK cell activation in response to viral infection can also result in a direct contribution to viral disease pathogenesis by mediating excessive tissue damage, as was described as possibly being the case in some select influenza viruses.

**Figure 8:**
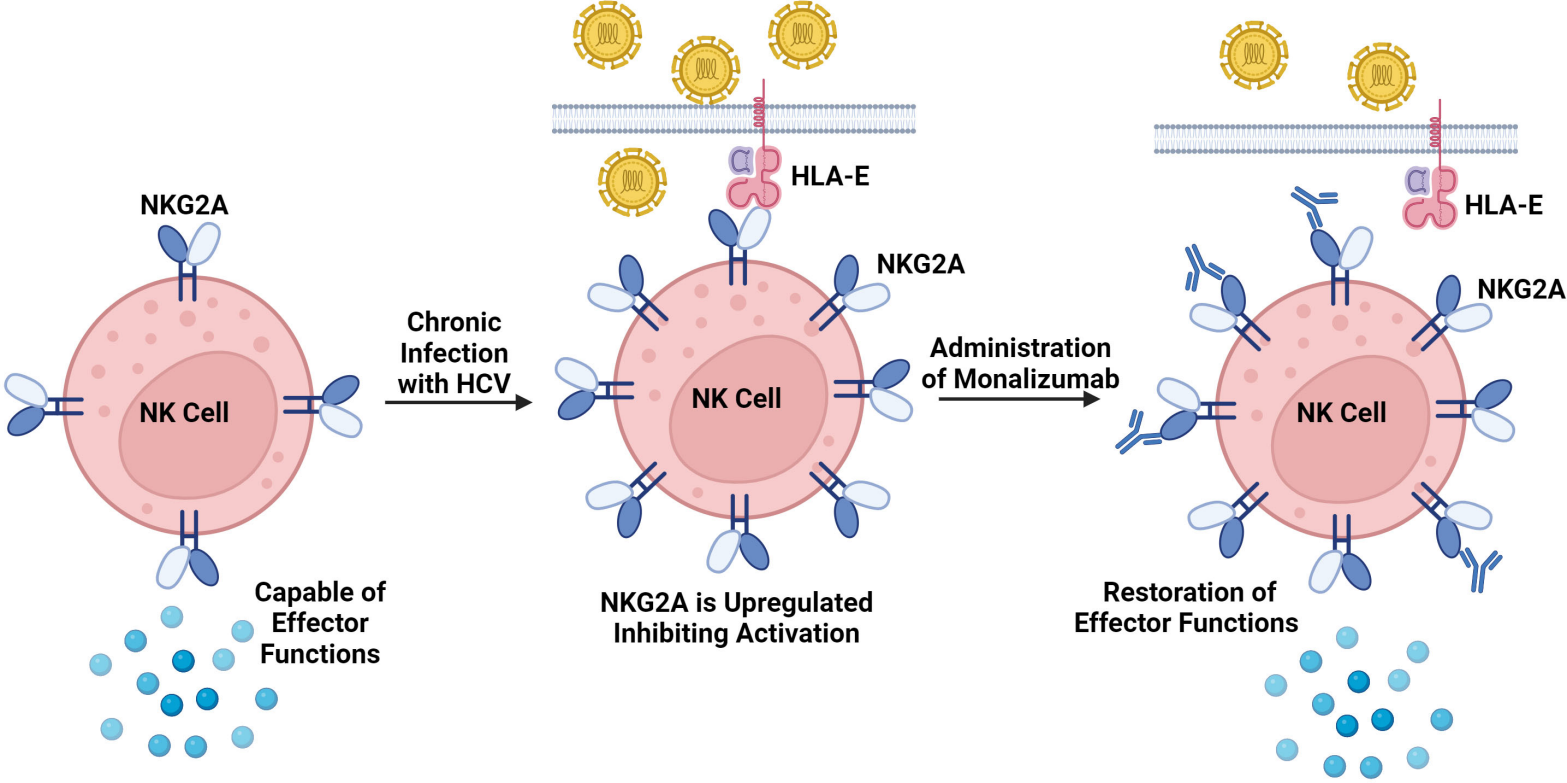
Schema of the Logic Underpinning the Use of NK Cell Therapeutics in Viral Infection. Many viral infections are associated with changes in the NK cell repertoire in terms of receptor expression and subset prevalence that result in altered NK cell functionality. Specific changes in NK cell repertoire can be targeted for therapeutic modulation to alter functionality in order to combat pathogenesis. For example, chronic HCV is associated with a significant increase in the expression of NKG2A, ultimately contributing to NK cell exhaustion, and a diminished antiviral response. The administration of NKG2A blockade interferes with inhibitory signaling in an effort to help restore effector functions to promote antiviral immunity.

While significant work has been done characterizing the NK cell repertoire correlated with disease state, less work has been done regarding elucidating the signaling environment that leads to changes in the NK cell population *in vivo*. As discussed, while cytokines like TGF-β are known to alter NK cell phenotype and function, the full landscape of signaling that leads to disease-specific NK cell repertoire requires more broad characterization. This rings especially true when considering the extensive genetic variability present in both HLA and important receptor families such as the KIR receptor family. The significant genetic variance in terms of NK cell repertoire and the subsequent corresponding differences in affinity between NK cell receptors and their ligands renders it extremely difficult to elucidate relationships between NK cell repertoire and disease. In essence, the state of an NK cell response, is determined not just by the relative expression of these receptors and their ligands, but by their genetic content and the relative receptor-ligand affinity it institutes. Despite these limitations, early preclinical and clinical work testing therapeutics seeking to address the changes in NK cell repertoire and activity associated with viral infection remains promising (**Figure 8**).

## Author contributions

JB: Conceptualization, Investigation, Project administration, Supervision, Writing – original draft, Writing – review & editing. JD: Writing – original draft, Writing – review & editing. RC: Writing – original draft, Writing – review & editing. GH: Writing – review & editing. JR: Conceptualization, Supervision, Writing – original draft, Writing – review & editing.
